# Anti-inflammatory potential of simvastatin and amfenac in ARPE-19 cells; insights in preventing re-detachment and proliferative vitreoretinopathy after rhegmatogenous retinal detachment surgery

**DOI:** 10.1007/s10792-024-03067-z

**Published:** 2024-03-26

**Authors:** Niina Harju, Maria Hytti, Onni Kolari, Hilkka Nisula, Sirpa Loukovaara, Anu Kauppinen

**Affiliations:** 1 School of Pharmacy, Faculty of Health Sciences, University of Eastern Finland, Kuopio, Finland; 2 Head and Neck Center, Ophthalmology Research Unit, Helsinki University Central Hospital, Helsinki, Finland; 3 Department of Ophthalmology, School of Medicine, Faculty of Health Sciences, University of Eastern Finland, Kuopio, Finland; 4 Department of Ophthalmology, Unit of Vitreoretinal Surgery, and Individualized Drug Therapy Research Program, Helsinki University Central Hospital and University of Helsinki, Helsinki, Finland

**Keywords:** Rhegmatogenous retinal detachment, RPE cells, Amfenac, Simvastatin, Inflammation, Proliferative vitreoretinopathy

## Abstract

**Purpose:**

Rhegmatogenous retinal detachment is a severe vision-threatening complication that can result into proliferative vitreoretinopathy (PVR) and re-detachment of the retina if recovery from surgery fails. Inflammation and changes in retinal pigment epithelial (RPE) cells are important contributors to the disease. Here, we studied the effects of simvastatin and amfenac on ARPE-19 cells under inflammatory conditions.

**Methods:**

ARPE-19 cells were pre-treated with simvastatin and/or amfenac for 24 h after which interleukin (IL)-1α or IL-1β was added for another 24 h. After treatments, lactate dehydrogenase release, 3-(4,5-dimethylthiazol-2-yl)-2,5-diphenyltetrazolium bromide (MTT) processing, nuclear factor kappa-light-chain-enhancer of activated B cells (NF-κB) activity, prostaglandin E2 (PGE2) level, and extracellular levels of IL-6, IL-8, monocytic chemoattractant protein (MCP-1), vascular endothelial growth factor (VEGF), and pigment epithelium-derived factor, as well as the production of reactive oxygen species (ROS) were determined.

**Results:**

Pre-treatment of human ARPE-19 cells with simvastatin reduced the production of IL-6, IL-8, and MCP-1 cytokines, PGE2 levels, as well as NF-κB activity upon inflammation, whereas amfenac reduced IL-8 and MCP-1 release but increased ROS production. Together, simvastatin and amfenac reduced the release of IL-6, IL-8, and MCP-1 cytokines as well as NF-κB activity but increased the VEGF release upon inflammation in ARPE-19 cells.

**Conclusion:**

Our present study supports the anti-inflammatory capacity of simvastatin as pre-treatment against inflammation in human RPE cells, and the addition of amfenac complements the effect. The early modulation of local conditions in the retina can prevent inflammation induced PVR formation and subsequent retinal re-detachment.

**Supplementary Information:**

The online version contains supplementary material available at 10.1007/s10792-024-03067-z.

## Introduction

Rhegmatogenous retinal detachment (RRD) surgery is one of the most common vitreoretinal operations, and ca. 10% of patients need re-surgery due to the re-detachment of the retina [1–3]. RRD can develop into proliferative vitreoretinopathy (PVR) if prolonged or if recovery from surgery fails [3, 4]. Inflammation and retinal pigment epithelial (RPE) cell changes are important contributors to the development of PVR i.e. the formation of scar tissue that predisposes to re-detachment of the retina [3, 4].

RPE cells are critical for the homeostasis of the retina and for the functionality and connection of photoreceptors to the choroid [4, 5]. In RRD, the retina breaks, and the vitreous fluid penetrates into the sub-retinal space i.e. between RPE cells and photoreceptors [6]. RPE cells begin to proliferate and migrate into the vitreous cavity and to the surface of the retina and after epithelial-mesenchymal transition (EMT), they participate in the formation of scar tissue under inflammatory conditions [3, 7, 8]. Interleukin (IL)-1 family cytokines are central pro-inflammatory mediators in retinal degenerative diseases, and RPE cells contribute to their production [9]. IL-1α is an early marker of inflammation and cell injury, and patients with retinal detachment have increased IL-1β levels in the subretinal fluid [9–12]. Cytokines indicate a risk for developing PVR [13–16]. For example, IL-1, IL-6, IL-8, and MCP-1 in the vitreous have been associated with retinal detachment and PVR [13–17].

Our clinical study revealed that a long-term systemic statin medication during the RRD surgery resulted in reduced risk of re-detachment and -operation [2]. The majority of patients used simvastatin that is a semi-synthetic hydroxymethylglutaryl (HMG)-CoA reductase inhibitor commonly used in cardiovascular diseases to reduce cholesterol formation [18]. Simvastatin has been shown to reduce the proliferation of primary human RPE cells and cells isolated surgically from PVR membranes [19]. Furthermore, the anti-inflammatory and immunomodulatory potential of statins has been acknowledged in several ocular disorders, such as uveitis, dry eye, and orbital and ocular adnexal inflammation [20–22]. In our previous study, statins alleviated lipopolysaccharide (LPS)-induced inflammation in ARPE-19 cells [20].

Topical administration of nonsteroidal anti-inflammatory drugs (NSAIDs, e.g., ketorolac and nepafenac) are efficient in alleviating inflammation in the anterior eye segment as well as in reducing the need of anti-vascular endothelial growth factor (anti-VEGF) or laser treatments for macular edema after a cataract surgery [23–27]. Nepafenac has several beneficial effects also at the posterior part of the eye, such as suppression of retinal prostaglandin production, protection of the blood-retina barrier, and prevention of concurrent protein extravasation into the vitreous [23]. Nepafenac is hydrolysed in intraocular tissues into amfenac, a cyclo-oxygenase (COX) inhibitor with potential to reduce inflammation and enter the posterior part of the eye [23–26].

Our main aim was to study whether simvastatin alone or together with amfenac has potential to prevent acute inflammation of RPE cells, which would be beneficial in preventing PVR formation and retinal re-detachment after RRD surgery. Our results suggest that early application of simvastatin together with amfenac has potential to alleviate the induction of inflammation in RPE cells indicating potential to modulate local conditions to prevent the development of PVR formation and re-detachment of the retina.

## Materials and methods

### Cells and treatments

All experiments were conducted using a commercial human RPE cell line [ARPE-19; American Type Culture Collection (ATCC)]. Cells were maintained in Dulbecco’s modified Eagle’s medium (DMEM/F-12 1:1; Life Technologies, Paisley, UK) with an antibiotic mixture of penicillin (100 U/ml) and streptomycin (100 μg/ml; Life Technologies, Grand Island, NY, USA), L-glutamine (2 mM, Life Technologies, Paisley, UK), and fetal bovine serum (FBS 10%, GE Healthcare Life Sciences, South Logan, UT, USA). Experiments were performed in serum-free DMEM/F-12 medium with L-glutamine and antibiotics. For thiazole blue/3-(4,5-dimethylthiazol-2-yl)-2,5-diphenyltetrazolium bromide (MTT), lactate dehydrogenase (LDH), interleukin (IL)-6, IL-8, monocytic chemoattractant protein (MCP-1), vascular endothelial growth factor (VEGF), and pigment epithelium-derived factor (PEDF) measurements, cells were seeded onto 12-well plate (Corning Incorporated Costar, Kennebunk, ME, USA) at 200 000 cells per well, for Nuclear factor kappa-light-chain-enhancer of activated B cells (NF-κB) and prostaglandin E2 (PGE2) measurements onto 6-well plate at 400 000 cells per well, and for reactive oxygen species (ROS) detection onto 96-well plate at 15 000 cells per well. Cells were cultured until confluency for three days in a humidified incubator at 5% CO_2_ and + 37 °C. Before exposures, cells were washed once with serum-free medium and then pre-treated with 5 μM simvastatin (Sigma Aldrich, USA) and/or 0.1 μM amfenac (Cayman Chemical, Michigan, USA) for 24 h in the incubator at 5% CO_2_, + 37 °C. Dimethyl sulfoxide (DMSO, 0.15%; Sigma Aldrich, St. Louis, MO, USA) was used as a diluent control for amfenac and simvastatin. Thereafter, recombinant human IL-1α (10 pg/ml; R&D Systems, Minneapolis, MN, USA) was added to cell cultures for additional 24 h excluding NF-κB and PGE2 measurement or ROS detection for which 4 h or 1 h incubations were applied, respectively. For ROS measurement, hydroquinone at 125 µM concentration was used as a positive control [28]. The release of LDH and cytokines (IL-6, IL-8, and MCP-1) were determined also after an exposure of cells to recombinant human IL-1β (5 pg/ml; Gibco, Carlsbad, CA, USA) for 24 h at 5% CO_2_, + 37 °C.

After treatments, cells were photographed under an Axio Vert A1 Zeiss microscope (Jena, Germany), after which medium samples were collected into microtubes (Sarstedt, Numbrecht, Germany), centrifuged (381×*g*, 10 min, + 4 °C), and stored at − 20 °C until analyses. Cells were purified from medium by adding Dulbecco’s phosphate-buffered saline (DPBS; Life Technologies, Paisley, UK) and scraped into microtubes with 200 µl DPBS, centrifuged (16 090×*g*, 10 min) and stored at − 80 °C. After thawing, cells were lysed by adding 60 μl of 1X lysis buffer (Cell lysis buffer 10X; Cell Signaling Technology, Leiden, Netherlands) and incubated for 5 min on ice. Thereafter, cell lysates were sonicated three times á 10 s and centrifuged at 16 090×*g* and + 4 °C for 10 min. Protein levels of the supernatants were determined using a method originated from the Bradford analysis, as described previously [29, 30].

### Cell viability

The cell membrane integrity was studied using the commercial CytoTox96 Non-Radioactive Cytotoxicity Assay (Promega, Madison, WI, USA) just after the sample collection avoiding the loss LDH enzyme activity due to freezing. Medium samples were collected as described above, and 50 μl per well was added onto a 96-well plate (Greiner Bio-One GmbH, Frickenhausen, Germany). Briefly, 50 μl of substrate mixture was added into each well and incubated with medium samples at room temperature for 30 min in darkness. Thereafter, stop solution (50 μl) was added, and absorbances were measured using a spectrophotometer (BioTek, ELx808; Instruments Inc., Winooski, VT, USA) at the 490 nm wavelength.

Mitochondrial activity of the cells was monitored using the MTT (Sigma-Aldrich, St. Louis, MO, USA) assay. After exposures, 500 μl medium was left in each well of the cell culture plates, MTT salt (10 mg/ml) in DPBS was added, and cells were incubated for 3 h at 5% CO_2_, + 37 °C. Thereafter, medium and MTT salt solution were replaced with 1000 μl of DMSO (Fischer Scientific, Leics, UK) and incubated for 20 min at room temperature. Thereafter, 200 μl of DMSO was transferred into wells of 96-well plate (Greiner Bio-One GmbH, Frickenhausen, Germany), and absorbance values were determined using the BioTek ELx808 spectrophotometer at the wavelength of 560 nm.

### Enzyme-linked immunosorbent assay

The DNA binding of NF-κB was measured from cell lysate samples with 15 μg of total protein in each sample using the commercial Active Motif TransAM NF-κB p65 (Carlsbad, CA, USA) assay. PGE2 was measured from cell lysates as pg/ml values using commercial Prostaglandin E2 Express Elisa kit (Cayman chemical, USA). Enzyme-linked immunosorbent assay (ELISA) analyses for cytokines IL-6, IL-8 and MCP-1 were performed from medium samples. IL-6 and IL-8 levels were detected using the BD OtpEIA™ Human ELISA sets (BD Biosciences, San Diego, CA, USA) and MCP-1 using the Human CCL2 (MCP-1) ELISA set (Thermo Fisher Scientific, San Diego, CA, USA). VEGF and PEDF were determined from medium samples using DuoSet Human VEGF and Human Serpin F1/PEDF DuoSet ELISA kits (both R&D Systems; Minneapolis, MN, USA). ELISA measurements were performed according to the manufacturers’ instructions and absorbance values were measured using a Bio-Rad Model 550 spectrophotometer (Bio-Rad Laboratories Inc., Hercules, CA, USA) at the wavelength of 450 nm with the correction wavelength of 655 nm.

### Intracellular ROS determination

After treatments, cells were washed once with serum-free medium. Thereafter, cells were exposed to the membrane permeable 2′,7′-dichlorodihydrofluorescein diacetate (H_2_DCFDA; 5 μM) probe (Molecular probes, Life Technologies, Eugene, USA) for 1 h at 5% CO_2_, + 37 °C. The probe is cleaved by ROS to 2′,7′-dichlorofluorescein (DCF) resulting in fluorescence emission. Hydroquinone (125 µM; Sigma-Aldrich, Saint Louis, MO, USA) was used as a positive control for ROS production [28]. After the probe incubation, cells were washed two times with 100 μl DPBS, 100 μl DPBS was added, and the fluorescence was measured using a fluorometer (BioTek Cytation3; Winooski, VT, USA) at the wavelengths of 488 nm and 528 nm (excitation/emission).

### Statistical analyses

Statistical analyses were performed using the GraphPad prism 7.04 software (San Diego, CA, USA). Multiple comparison between groups was performed using the Kruskal–Wallis test and pairwise comparisons using the Mann–Whitney U test. Results were considered as statistically significant at *P*-values lower than 0.05. The data are presented as mean ± standard error of mean (SEM).

## Results

### Simvastatin and amfenac are well tolerated by RPE cells upon IL-1α exposure

**Fig. 1:**
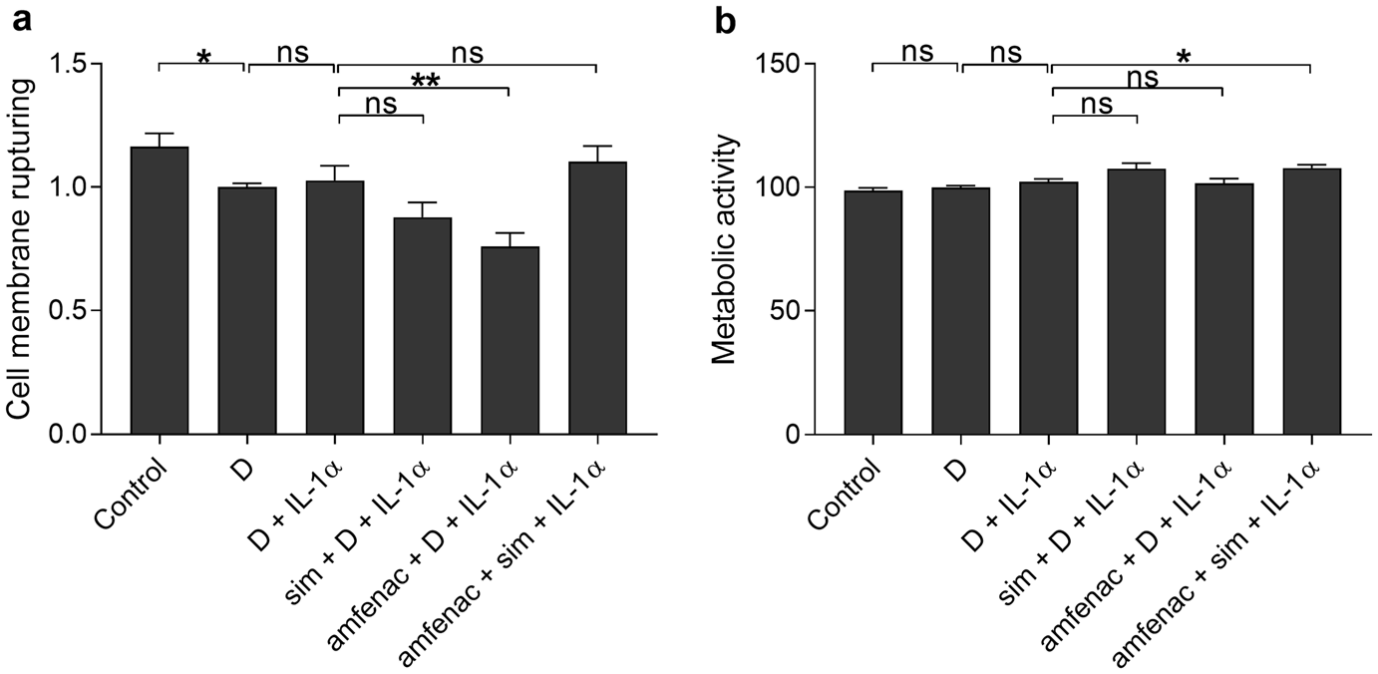
The effects of simvastatin (sim; 5 µM) and amfenac (0.1 µM) on the LDH release (**a**) and metabolic activity measured using the MTT assay (**b**) in IL-1α-treated (10 pg/ml) ARPE-19 cells. Results were normalized to the DMSO control (D; 0.15%). Data were collected from 3 independent experiments including 4 samples per group in each experiment (total n = 12), and results are presented as mean ± standard error of mean (SEM). *P < 0.05, **P < 0.01, ns—not significant by Mann–Whitney U test. (Kruskal–Wallis test was performed but not presented in the Figure, **a** ****P < 0.0001, **b** **P < 0.01)

**Fig. 2:**
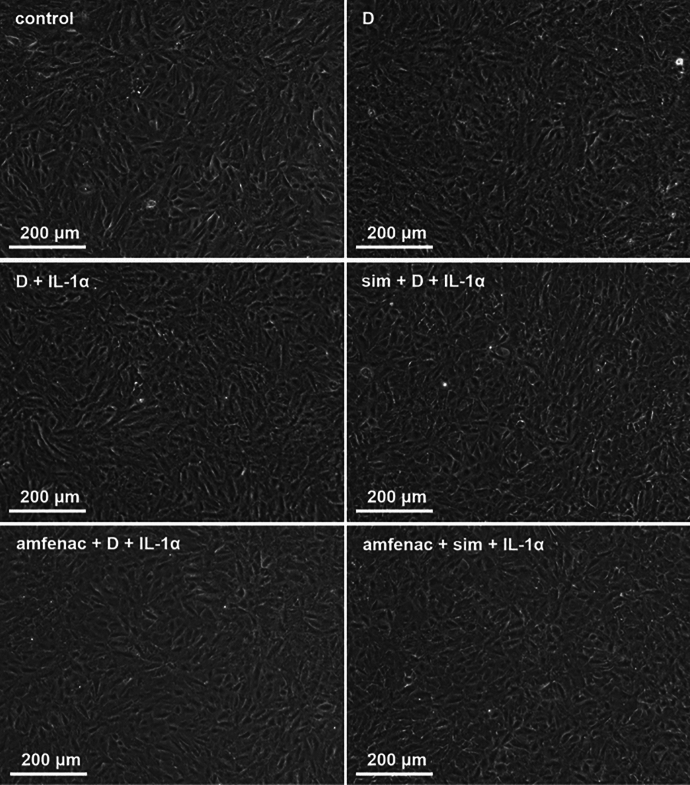
The effect of simvastatin (sim; 5 µM) and amfenac (0.1 µM) on IL-1α-treated (10 pg/ml) ARPE-19 cells. Cells were photographed under an inverted phase contrast light microscope Axio Vert A1 Zeiss. (D, DMSO; 0.15%)

IL-1 cytokines are critical contributors to inflammatory and degenerative diseases in the retina [9]. The selected IL-1α concentration has been associated with mild inflammation in the eye, such as in corneal epithelial erosion patients [31, 32]. Simvastatin concentration is based on our previous study where it reduced inflammation in RPE cell cultures upon exposure to lipopolysaccharide (LPS) in ARPE-19 cells [20], and amfenac concentration was selected based on literature [25, 33] and preliminary results (Supplementary Figs. 1, 2). IL-1α did not affect the cell membrane integrity or metabolic activity of ARPE-19 cells when compared to cells exposed only to DMSO, which was used as a solvent for simvastatin and amfenac (Fig. 1). The used DMSO volume was not harmful for the cells (Fig. 1). The addition of simvastatin to IL-1α-treated cells neither caused changes in cell viability (Fig. 1). Instead, amfenac reduced LDH release and did not change metabolic activity of the cells when compared to cells exposed only to IL-1α and DMSO (Fig. 1). Together, simvastatin and amfenac increased metabolic activity in RPE cells without changing the cell membrane integrity when compared to IL-1α and DMSO -treated cells (Fig. 1). Microscopic analysis supported good condition of IL-1α-treated cells upon exposure either to simvastatin, amfenac, or both (Fig. 2).

### Simvastatin reduces the levels of active NF-κB in ARPE-19 cells

**Fig. 3:**
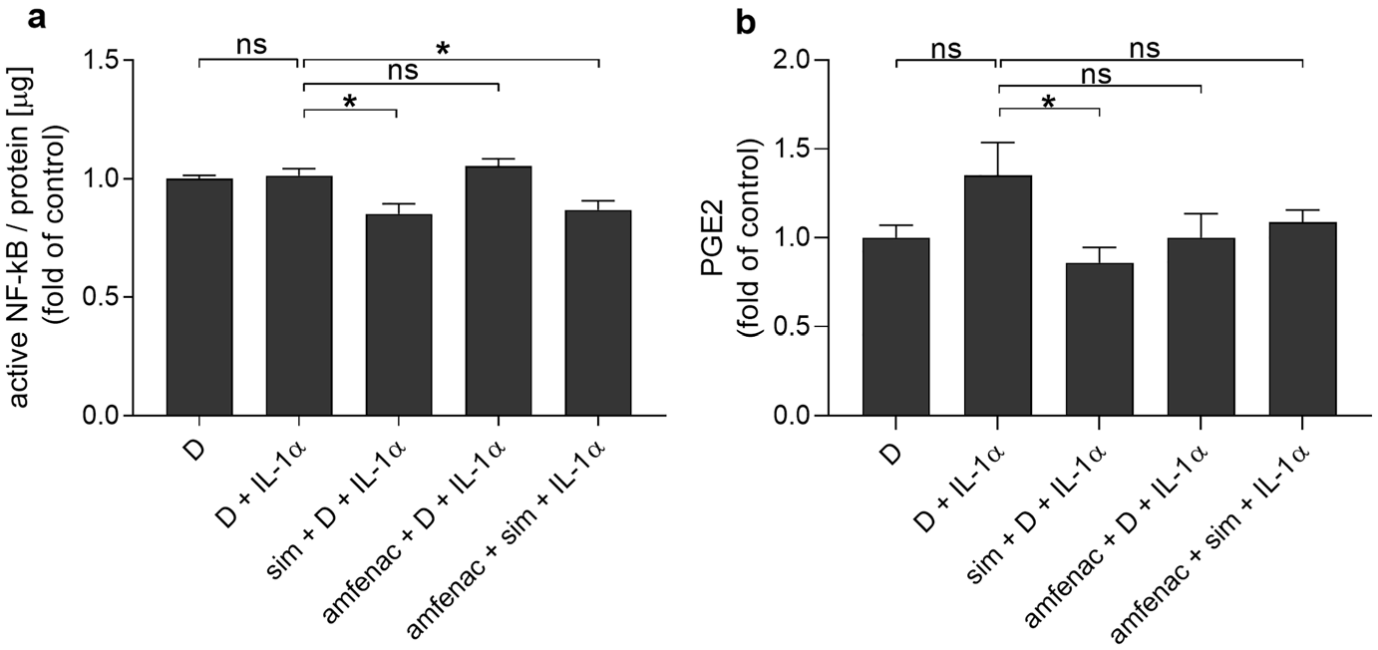
The effects of simvastatin (sim; 5 µM) and amfenac (0.1 µM) on the levels of active NF-κB p65 (**a**) and PGE2 (**b**) in ARPE-19 cells upon IL-1α (10 pg/ml) exposure. NF-κB results were normalized to the protein level of each sample and compared to the mean of DMSO group (D; 0.15%). PGE2 levels were measured from cell lysates as pg/ml values and compared to the mean of DMSO-treated control cells. Data were collected from 3 independent experiments including 2–3 samples per group in each experiment (total n = 8), and results are presented as mean ± SEM. *P < 0.05, ns—not significant by Mann–Whitney U test. [Kruskal–Wallis test was performed but not presented in the Figure, ***P < 0.001 (**a**), ns—not significant (**b**)]

Since inflammation contributes to the development of PVR after unsuccessful recovery from RRD surgery [3], we wanted to measure the effect of simvastatin and amfenac on the activity of NF-κB that regulates the production of many pro-inflammatory cytokines. IL-1α exposure did not significantly increase NF-κB levels when compared to cells exposed to DMSO (Fig. 3a). We have previously shown that DMSO does not alter the NF-κB activity in comparison to untreated cells [28]. Simvastatin alone or together with amfenac significantly reduced the DNA binding of NF-κB p65 whereas amfenac had no effect (Fig. 3a). Since prostaglandin and NF-κB regulate each other and NSAIDs are known COX-inhibitors [34–36], we measured also PGE2 levels. The used IL-1α concentration did not change PGE2 levels in ARPE-19 cells when compared to DMSO-treated control cells (Fig. 3b). Instead, simvastatin alone reduced PGE2 levels in comparison to IL-1α but not when combined with amfenac (Fig. 3b). Amfenac alone did not reduce intracellular PGE levels. These data suggest that simvastatin has potential to regulate NF-κB activation and PGE2 levels in human RPE cells upon IL-1α exposure.

### Simvastatin and amfenac reduce the release of pro-inflammatory cytokines from ARPE-19 cells upon exposure to IL-1α or IL-1β

**Fig. 4:**
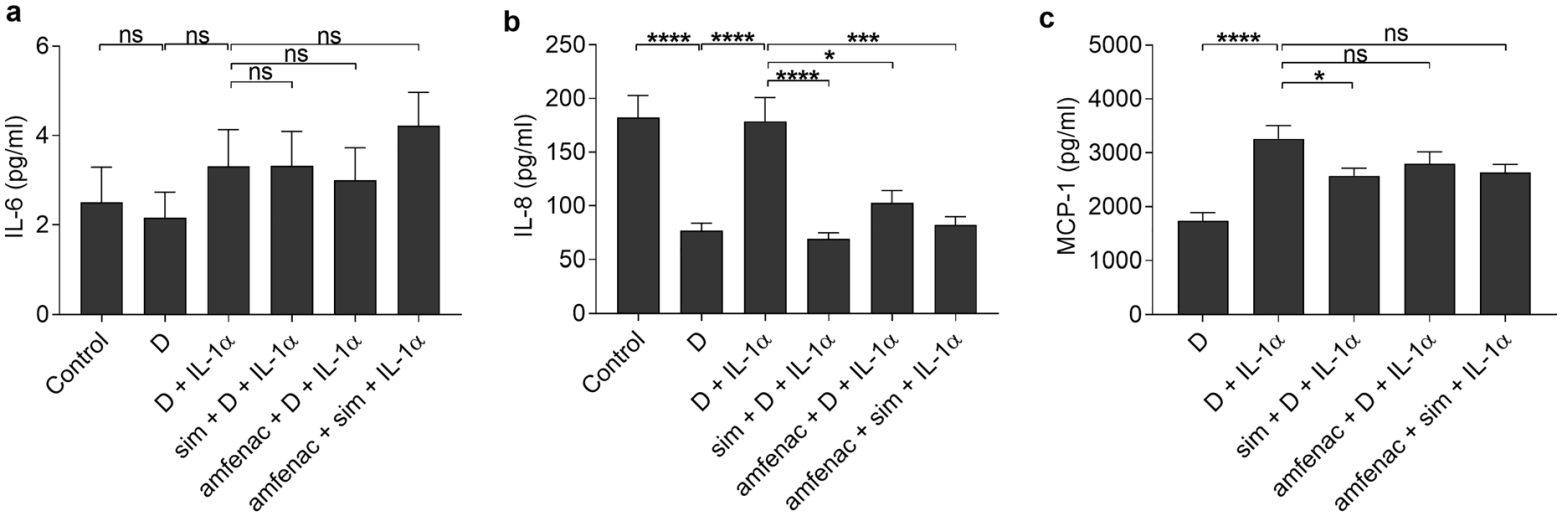
The effects of simvastatin (sim; 5 µM) and amfenac (0.1 µM) on the release of cytokines IL-6 (**a**), IL-8 (**b**), and MCP-1 (**c**) from IL-1α (10 pg/ml) -treated ARPE-19 cells. DMSO (D; 0.15%) was used as a solvent control for simvastatin and amfenac. Data were collected from 3 independent experiments including 4 (**a**, **b**) or 2–4 (**c**) samples per group in each experiment [total n = 12 (**a**, **b**) and n = 10 (**c**)], and results are presented as mean ± SEM. *P < 0.05, ***P < 0.001, ****P < 0.0001, ns—not significant by Mann–Whitney U test. (Kruskal–Wallis test was performed but not presented in the Figure, **a** not significant, **b** ****P < 0.0001, **c** ***P < 0.001)

Since the regular systemic use of statins prior to surgery has been shown to reduce the need of re-surgery for retinal detachment, and topical NSAIDs reduce macular edema after cataract surgery [2, 37], we wanted to study whether simvastatin and/or amfenac have direct anti-inflammatory effects on RPE cells. We have previously shown that 0.5% DMSO reduces the release of cytokines (IL-6, IL-8, and MCP-1) when compared to untreated control cells [28]. DMSO has anti-inflammatory features e.g. by reducing cytokine production as well as the levels of prostaglandin and mitogen-activated protein kinases (MAPKs) [38]. In the presence of IL-1α, 0.15% DMSO reduced IL-8 levels (Fig. 4). The used IL-1α concentration alone did not induce IL-6 but increased IL-8 and MCP-1 production by ARPE-19 cells (Fig. 4). Neither simvastatin nor amfenac had effect on IL-6 levels when compared to cells treated by IL-1α without additional exposure. Simvastatin alone reduced the release of IL-8 and MCP-1, whereas amfenac with or without simvastatin reduced IL-8 but had no effect on MCP-1. Collectively, these data suggest that simvastatin and amfenac have potential to reduce the release of chemoattractant proteins from human RPE cells, both may reduce active inflammation by reducing the chemotaxis of neutrophils [39], and simvastatin can also reduce the attraction of monocytes to the retina [40].

**Fig. 5:**
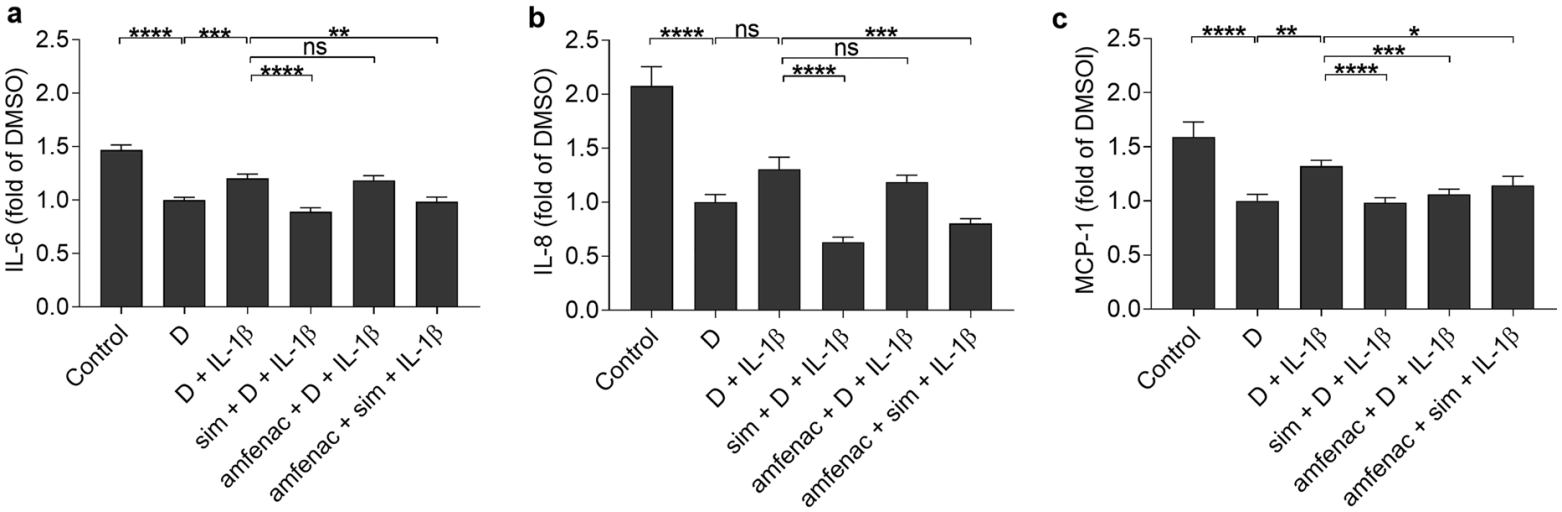
The effects of simvastatin (sim; 5 µM) and amfenac (0.1 µM) on the release of cytokines IL-6 (**a**), IL-8 (**b**), and MCP-1 (**c**) from IL-1β-treated (5 pg/ml) ARPE-19 cells. DMSO (D; 0.15%) was used as a solvent control to simvastatin and amfenac. Data were collected from 3 independent experiments including 4 samples per group in each experiment (total n = 12) and results are presented as mean ± SEM. *P < 0.05, **P < 0.01, ***P < 0.001, ****P < 0.0001, ns—not significant by Mann–Whitney U test. (Kruskal–Wallis test was performed but not presented in the Figure, **a** ****P < 0.0001, **b** ****P < 0.0001, **c** ****P < 0.0001)

**Fig. 6:**
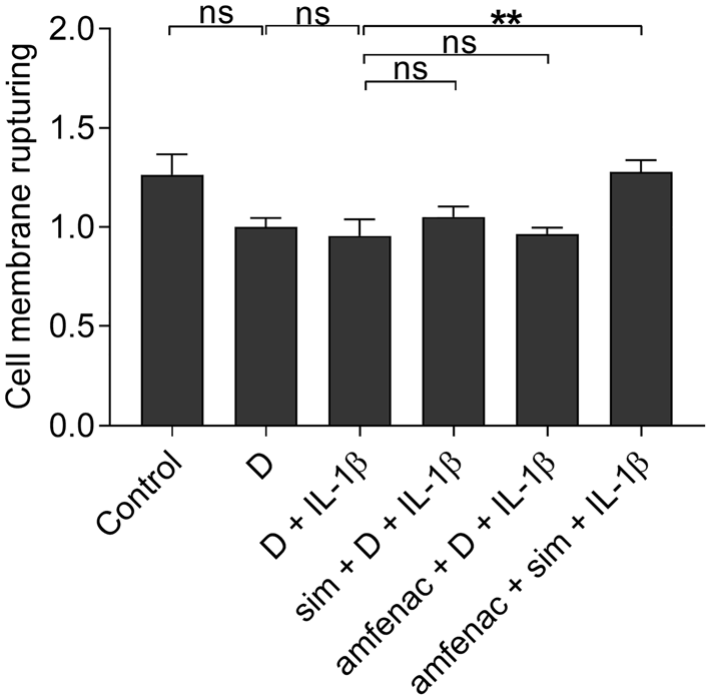
The effects of simvastatin (sim; 5 µM) and amfenac (0.1 µM) on the release of LDH from IL-1β treated (5 pg/ml) ARPE-19 cells. DMSO (D; 0.15%) was used as a solvent control to simvastatin and amfenac. Data were collected from 3 independent experiments including 4 samples per group in each experiment (total n = 12) and results are presented as mean ± SEM. **P < 0.01, ns—not significant by Mann–Whitney U test. (Kruskal–Wallis test was performed but not presented in the Figure, **P < 0.01)

**Fig. 7:**
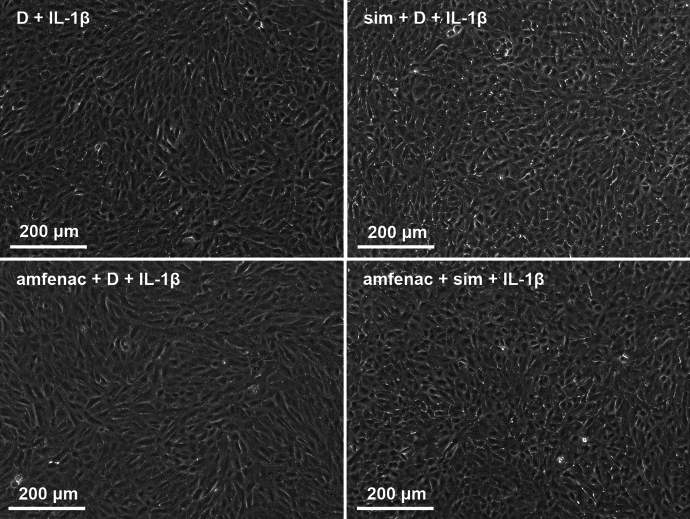
The effect of simvastatin (sim; 5 µM) and amfenac (0.1 µM) on IL-1β-treated (5 pg/ml) ARPE-19 cells

Since IL-1β production is an important contributor in early retinal detachment [10, 41], we tested the anti-inflammatory capacity of simvastatin and amfenac also upon IL-1β exposure of RPE cells. In the presence of IL-1β, DMSO reduced the release of IL-6, IL-8, and MCP-1 cytokines when compared to untreated cells (Fig. 5). IL-1β induced the production of IL-6 and MCP-1 (Fig. 5). The pre-treatment of cells with simvastatin alone or together with amfenac significantly reduced the release of all three cytokines (Fig. 5). Amfenac alone significantly reduced the MCP-1 secretion but had no effect on the release of IL-6 or IL-8 when compared to IL-1β-treated RPE cells. Together simvastatin and amfenac compromised cell viability seen as significantly increased LDH release but separately both were well tolerated (Fig. 6). However, visible damage was not present in cell images (Fig. 7). Taken together, our present data allude that simvastatin could reduce the levels of chemokines that recruit neutrophils and monocytes to the inflamed tissue [39, 40, 42], whereas amfenac has slightly narrower capacity to prevent the release of chemokines. It is noteworthy that simvastatin also reduced IL-6 levels, which is critical factor for the EMT process in RPE cells as well as for the PVR formation [13, 14, 17].

### Increased ROS production by simvastatin and amfenac is combined with reduced cytokine release in ARPE-19 cells

**Fig. 8:**
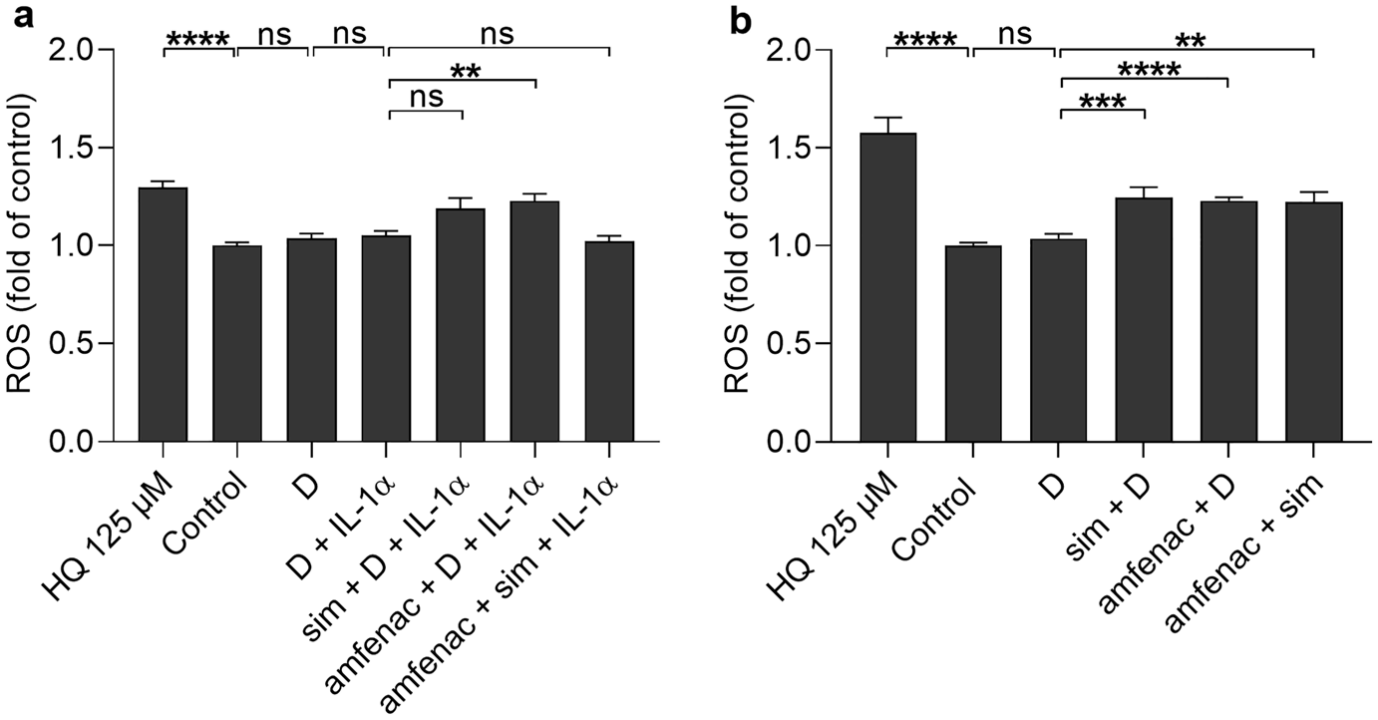
The effects of simvastatin (sim; 5 µM) and amfenac (0.1 µM) on the levels of intracellular ROS with (**a**) or without **(b)** 10 pg/ml IL-1α in ARPE-19 cells. DMSO (D; 0.15%) was used as dilution control of simvastatin and amfenac, and hydroquinone (HQ) 125 µM as a positive control for ROS production. Data were collected from 3 independent experiments containing 6 samples per group in each experiment (total n = 18), and results are presented as mean ± SEM. **P < 0.01, ***P < 0.001, ****P < 0.0001, ns—not significant by Mann–Whitney U test. (Kruskal–Wallis test was performed but not presented in the Figure, **a** ****P < 0.0001, **b** ****P < 0.0001)

ROS production increased during induced retinal detachment in a rat model, whereas its reduction improved the photoreceptor viability [43]. On the other hand, low level of ROS is produced as well in normal conditions but its excessive amount is detrimental e.g. induces inflammation [44], for which we took the effect of simvastatin and amfenac on the ROS production into consideration, as well. IL-1α alone or together with simvastatin or both simvastatin and amfenac, had no effect on the ROS production (Fig. 8a). Instead, amfenac increased intracellular ROS levels upon IL-1α-induced inflammation (Fig. 8a). In non-inflammatory conditions, simvastatin and amfenac alone and together increased ROS production in ARPE-19 cells (Fig. 8b). Collectively, amfenac and simvastatin showed properties to increase ROS levels in non-inflammatory and in induced inflammatory conditions in ARPE-19 cells although the production of pro-inflammatory cytokines was reduced (Fig. 4).

### Simvastatin together with amfenac increases VEGF release from ARPE-19 cells

**Fig. 9:**
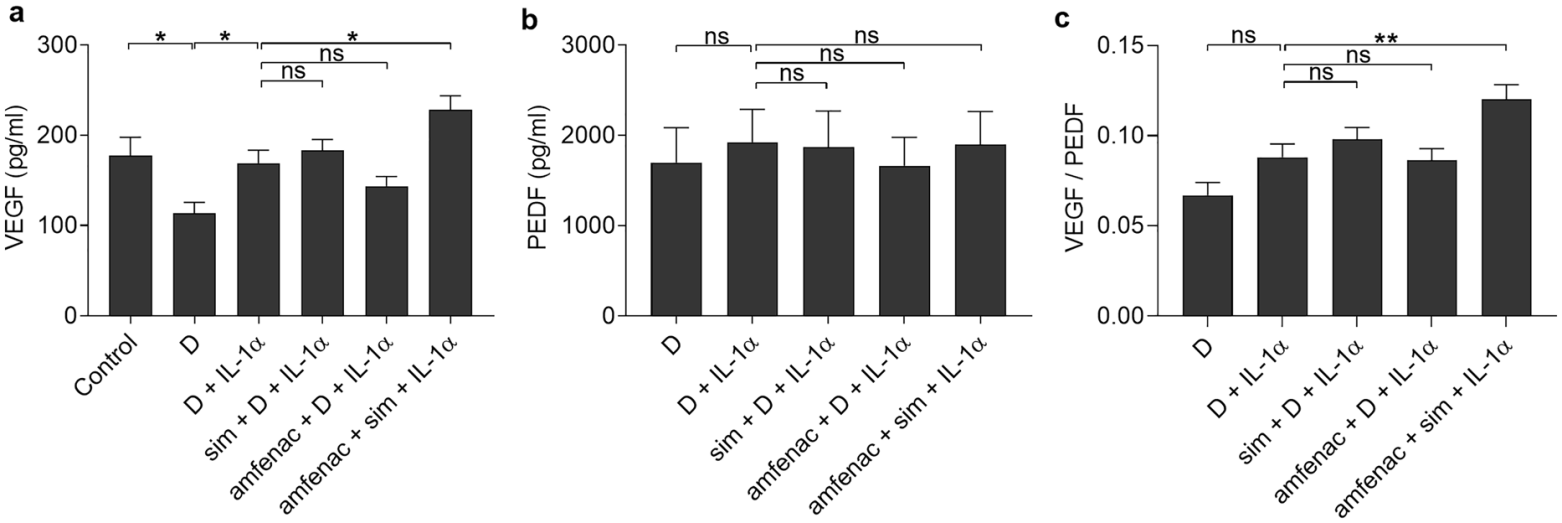
The effects of simvastatin (sim; 5 µM) and amfenac (0.1 µM) on the release of VEGF (**a**) and PEDF (**b**), and on the ratio of VEGF with PEDF (**c**) in 10 pg/ml IL-1α treated ARPE-19 cells. DMSO (D; 0.15%) was used as dilution control of simvastatin and amfenac. Data were collected from 3 independent experiments containing 4 (**a**, **c**) and 2 (**b**, **c**) samples per group in each experiment [total n = 12 (**a**, **c**) and n = 6 (**b**, **c**)], and results are presented as mean ± SEM. *P < 0.05, **P < 0.01, ns—not significant by Mann–Whitney U test. (Kruskal–Wallis test was performed but not presented in the Figure, **a** ***P < 0.001, **b** not significant, **c** ***P < 0.001)

Since topical NSAID administration has been shown to reduce the need of anti-VEGF treatment for macular edema after cataract surgery [27], we tested whether simvastatin and/or amfenac has direct effects on the VEGF production of RPE cells under inflammatory conditions [45]. In addition, we measured the levels of PEDF, an anti-angiogenic factor that regulates VEGF [46, 47]. IL-1α (10 pg/ml) increased the VEGF release from RPE cells when compared to those exposed solely to the solvent control DMSO (Fig. 9a). Simvastatin or amfenac alone had no effect on the VEGF release from RPE cells after IL-1α exposure, whereas their combination significantly increased VEGF secretion. Neither IL-1α, simvastatin, nor amfenac changed PEDF levels (Fig. 9b). The VEGF/ PEDF ratio increased in IL-1α-treated ARPE-19 cells with the combined application of simvastatin and amfenac when compared to cells exposed only to IL-1α (Fig. 9c). Collectively, simvastatin together with amfenac had potential to increase VEGF production without any change in the PEDF release from RPE cells upon inflammatory conditions.

## Discussion

Proliferative vitreoretinopathy (PVR) can develop after RRD if recovery from surgery does not go as expected [3]. Inflammation and RPE changes are critical contributors to the disease progression. IL-1α is an early pro-inflammatory cytokine that promotes inflammation and necroptosis [11]. Macrophages are a major source of IL-1β in the subretinal space, but RPE cells secrete it, as well [10, 12, 41]. Macrophages infiltrating into the subretinal space promote the separation of RPE cells from the neuroretina and exacerbate the disease progression [48]. Pro-inflammatory cytokines IL-6, IL-8, MCP-1, as well as VEGF have been detected in the vitreous of retinal detachment patients [10, 49, 50]. In the present study, IL-6 production was increased by IL-1β in human RPE cells, and simvastatin alone or together with amfenac reduced it. Although IL-6 is important for the photoreceptor survival during retinal detachment, it is also a critical factor for the EMT process in RPE cells and its levels are increased in the vitreous of PVR patients [13, 14, 17, 51]. Both simvastatin and amfenac were able to reduce the chemokine IL-8 and MCP-1 release in the presence of either IL-1α or IL-1β. The pre-treatment of RPE cells with simvastatin reduced the activation of NF-κB upon IL-1α exposure but the effects of amfenac were mediated by another route. It has been proposed that the targeted reduction of chemokines reduces the rate of retinal detachment and improves the survival of photoreceptors [48]. The restriction of IL-8 and MCP-1 by simvastatin and/or amfenac could bring the same benefits since those chemokines are responsible for the infiltration of leukocytes from the circulation [39, 40]. Especially the reduction of MCP-1 is notable since it has been shown to be increased in the vitreous of RRD patients that are prone to develop PVR [52]. Simvastatin reduced the PGE2 levels, which is in line with reduced MCP-1 release since PGE2 induces MCP-1 expression [53, 54]. Our experimental model simulating the inflammatory conditions after retinal detachment surgery in RPE cells suggests that simvastatin and amfenac have potential to lower the risk for inflammation-related (Table 1) recurrent RRD or development to PVR.

**Table 1:** Combined effects of simvastatin and amfenac on IL-1α (10 pg/ml) or IL-1β (5 pg/ml)-induced inflammatory responses in human ARPE-19 cell cultures

Exposure	IL-1α	IL-1α	IL-1α			IL-1β		
Measurement	NF-κB	PGE2	IL-6	IL-8	MCP-1	IL-6	IL-8	MCP-1
Simvastatin	↓	↓	–	↓	↓	↓	↓	↓
Amfenac	–	–	–	↓	–	–	–	↓
Amfenac + simvastatin	↓	–	–	↓	–	↓	↓	↓

Detachment of photoreceptors from the RPE and the choroid results in immediate hypoxia and subsequently, damaged RPE cells produce ROS in the retina [43]. Roh et al. showed that the reduction of ROS production by edaravone during retinal detachment concurrently reduced inflammation and improved photoreceptor viability [43]. In the present study, simvastatin and amfenac increased ROS production, which could be a beneficial response to prevent early oxygen deficiency during retinal detachment [55–57]. It is noteworthy that concurrently with increased ROS, simvastatin and amfenac reduced inflammation. Further studies are needed to find out whether ROS production prevails upon prolonged exposure, which could cause opposite effects [57]. Reduced ROS levels could be beneficial at the later stage of retinal detachment when ROS levels increase to purely detrimental levels [43].

VEGF increased during the combination treatment of RPE cells with simvastatin and amfenac. That could improve the connection of RPE with photoreceptors since VEGF is important for the integrity of the retina [45]. The beneficial effect is supported by the observations that anti-VEGF treatment can contribute to tractional retinal detachment [58]. On the other hand, increased VEGF levels are associated with pathological neovascularization in the retina [45, 59, 60]. Increased VEGF levels in the vitreous of retinal detachment patients have been suggested to predispose to the development of PVR [50]. However, it is not clear whether increased VEGF levels after combination treatment would be harmful or beneficial since the effect is partially influenced also by the physiology of the subject. The highest VEGF levels have been detected in diabetic retinopathy-related retinal detachment where VEGF production can be associated also with the primary disease [49, 50]. Since simvastatin and amfenac together increased VEGF with no effect on the levels of PEDF, an important regulator of VEGF [46], our data allude that an exposure of RPE cells to both simvastatin and amfenac support the retinal integrity at low physiological VEGF levels but can be detrimental during neovascularization. The result of increased VEGF and PEDF ratio upon exposure of RPE cells to simvastatin and amfenac supports this hypothesis. Ischemia-induced damage in the retina has been shown to lead to increased VEGF/PEDF ratio and neovascularization [61]. Keeping that in mind, it could be advantageous that PEDF would increase along with VEGF. It is also positive that PEDF levels do not decrease upon exposure of RPE cells to simvastatin and amfenac since PEDF is needed to prevent apoptosis and suppress cytokine expression in the degenerating retina [47].

In conclusion, our present study suggests that well-tolerated simvastatin and amfenac have potential to modulate local conditions in the retina to prevent PVR formation and subsequent retinal re-detachment by reducing the formation of inflammation in RPE cells. According to our measurements, statin concentration levels in the vitreous of vitrectomy patients ranged from 0.01 to 0.7 nM [20]. As we have seen, already oral intake of simvastatin has beneficial effects in the eye [2]. The present study supports the aim to develop a routine to administrate simvastatin directly into the eye upon surgery for which the effects and safety of higher concentrations also deserve studying. It has been estimated that 0.8–6.4 mg of simvastatin is needed for local zero-order release loading dose and 7.1–59 mg for the first-order release loading dose to achieve 5 µM simvastatin concentration in the retina [20]. These are simulation-based calculations and real practical safety assessment is further needed.

## Supplementary Information

Below is the link to the electronic supplementary material.

Supplementary file1 (DOCX 359 KB)

## Data Availability

The data presented in this study are available on request from the corresponding authors.
