# Supplementary material for: Anti-inflammatory potential of simvastatin and amfenac in ARPE-19 cells; insights in preventing re-detachment and proliferative vitreoretinopathy after rhegmatogenous retinal detachment surgery
Source: Int Ophthalmol. 2024 Mar 26;44(1):158. doi: 10.1007/s10792-024-03067-z (PMC10965607; doi:10.1007/s10792-024-03067-z)
Supplement: Supplementary file 1 — Supplementary file1 (DOCX 359 KB) [file 10792_2024_3067_MOESM1_ESM.docx]

**Supplementary Data**


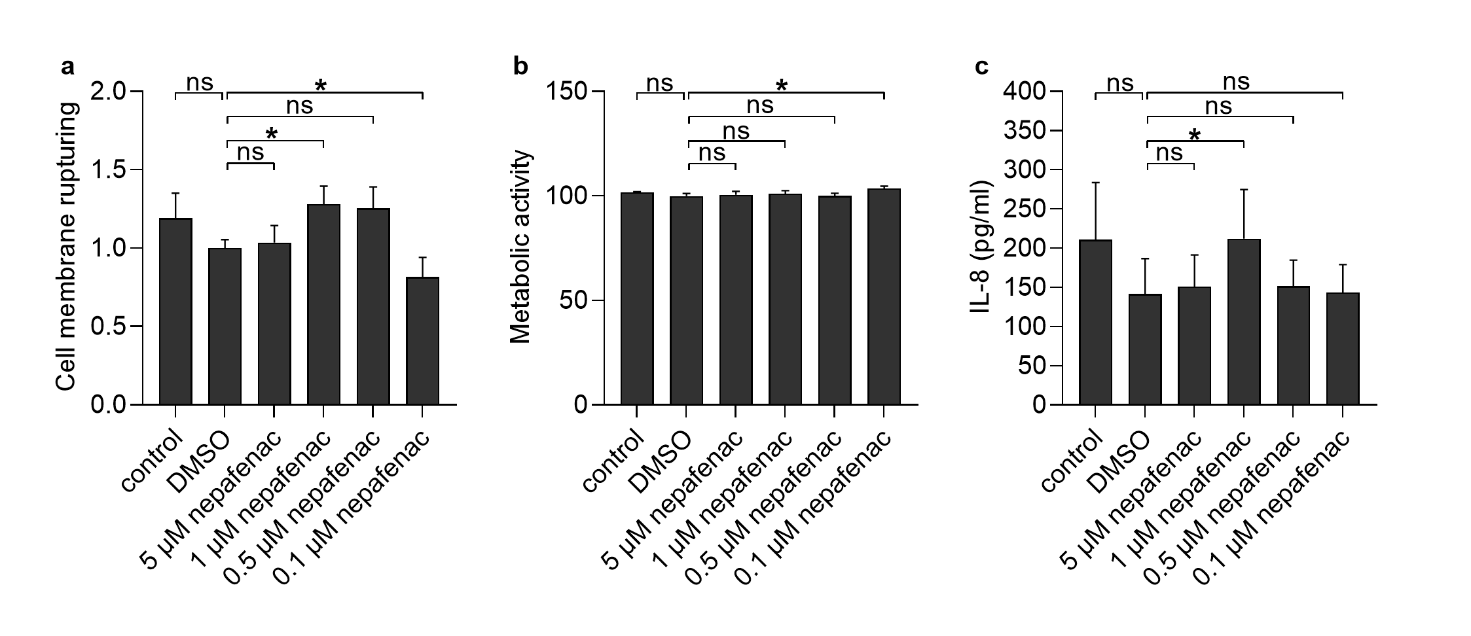


**Supplementary Figure 1**.The effect of nepafenac on the LDH release (a), metabolic activity of mitochondria (b), and the release of IL-8 (c) in ARPE-19 cells. Data were collected from 3 independent experiments including 4 samples per group in each experiment (total n=12), and results are presented as mean ± standard error of mean (SEM). * P < 0.05, ns – not significant by Mann-Whitney U test.


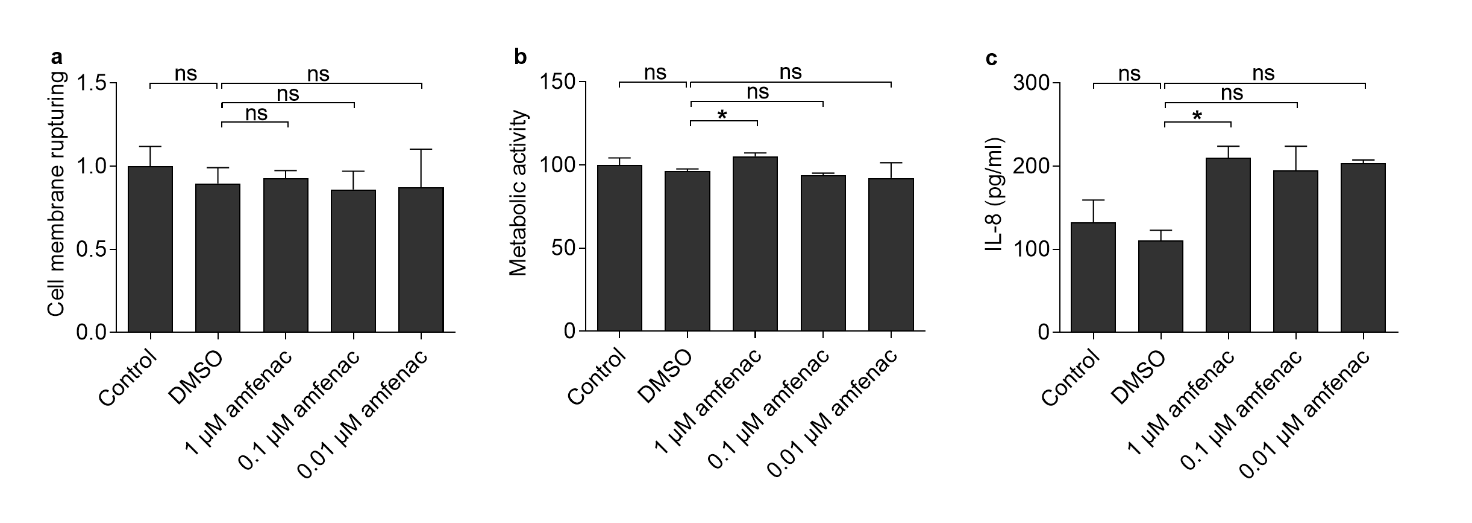


**Supplementary Figure 2**. The effect of amfenac on the LDH release (a), metabolic activity of mitochondria (b), and the release of IL-8 (c) in ARPE-19 cells. Data were collected from 1 independent experiment including 4 samples per group in each experiment (total n=4), and results are presented as mean ± standard error of mean (SEM). * P < 0.05, ns – not significant by Mann-Whitney U test.
